# Two-Tiered Ambulance Dispatch and Redeployment considering Patient Severity Classification Errors

**DOI:** 10.1155/2019/6031789

**Published:** 2019-12-09

**Authors:** Seong Hyeon Park, Young Hoon Lee

**Affiliations:** Department of Industrial Engineering, Yonsei University, D1010, 50, Yonsei-ro, Seodaemun-gu, Seoul, Republic of Korea

## Abstract

A two-tiered ambulance system, consisting of advanced and basic life support for emergency and nonemergency patient care, respectively, can provide a cost-efficient emergency medical service. However, such a system requires accurate classification of patient severity to avoid complications. Thus, this study considers a two-tiered ambulance dispatch and redeployment problem in which the average patient severity classification errors are known. This study builds on previous research into the ambulance dispatch and redeployment problem by additionally considering multiple types of patients and ambulances, and patient classiﬁcation errors. We formulate this dynamic decision-making problem as a semi-Markov decision process and propose a mini-batch monotone-approximate dynamic programming (ADP) algorithm to solve the problem within a reasonable computation time. Computational experiments using realistic system dynamics based on historical data from Seoul reveal that the proposed approach and algorithm reduce the risk level index (RLI) for all patients by an average of 11.2% compared to the greedy policy. In this numerical study, we identify the influence of certain system parameters such as the percentage of advanced-life support units among all ambulances and patient classiﬁcation errors. A key finding is that an increase in undertriage rates has a greater negative effect on patient RLI than an increase in overtriage rates. The proposed algorithm delivers an efficient two-tiered ambulance management strategy. Furthermore, our findings could provide useful guidelines for practitioners, enabling them to classify patient severity in order to minimize undertriage rates.

## 1. Introduction

Ambulance operating methods are highly important for the emergency medical service (EMS) system as they directly affect the patient survival rate and medical service quality. Two types of decision are required during ambulance operations: (1) the dispatch decision, i.e., which ambulance to send to an emergency call, and (2) the redeployment decision, i.e., the waiting location to which the ambulance that has just completed a patient-transport service should be sent. The goal of ambulance operations is to provide patients with appropriate emergency treatment within a short time period and then transport the patient to the hospital for specific advanced treatment. Therefore, an efficient strategy is required for dispatching and redeploying ambulances.

Emergency care and transport of patients should be both highly flexible and rapid because small time delays might have a negative impact on emergency patients. However, in an EMS system where patient numbers are highly uncertain, preplanned scheduling or operation solutions may not optimally respond to fluctuating situations. Therefore, real-time decision-making is required, which must consider system dynamics such as time-varying demands (emergency calls), time-varying traffic, and the different first-aid times required by patients. Another important consideration in ambulance operations is the different severity of the transported patients. The majority of patients are nonemergency patients. They request an ambulance because of a lack of transportation, inability to ambulate, domestic violence, or poor social situations while a few of them can either walk or use public transport to reach a hospital [1, 2]. Transfer of nonemergency patients by ambulance can be delayed due to the preferential transfer of emergency patients because their deterioration rate of health may be much lower. However, as only limited information is delivered during calls to the emergency operator, it is risky to designate a patient's severity as low and delay the dispatch of an ambulance to the patient. Therefore, all emergency calls must be responded to immediately regardless of the classified severity of patients; in South Korea, it is regulated by law.

Based on the criteria used in South Korea, ambulances are classified into two types based on the patients' level of urgency [3]. (1) An advanced life support (ALS) vehicle is suitable for emergency-patient transport. It must be accompanied by paramedics who can perform more specialized medical care and is designed with more stringent standards, including the minimum area for the patient in the ambulance and the medical equipment to be installed inside. (2) A basic life support (BLS) vehicle is suitable for nonemergency patient transport. It provides basic medical services with relatively little medical equipment and is accompanied by emergency medical technicians (EMTs). Therefore, high-risk emergency patients transported by BLS units would be at risk because they may not receive adequate care during transport. The corresponding ambulance systems are also classified into two types: an “all-ALS system” that operates all ambulances as ALS vehicles and a “two-tiered ambulance system (tiered system)” that uses a combination of ALS and BLS units. Previous research has debated the superiority of all-ALS or mixed-ALS/BLS ambulance management systems according to their relative risks, treatment times, and cost effectiveness [4–9].

To operate a two-tiered ambulance system efficiently, an emergency center should attempt to classify the severity of the patients during the emergency call. However, the lack of information obtained from the call inevitably leads to patient severity classification errors, which could have a devastating impact on the patient risk level. However, although previous research has attempted to optimize ambulance dispatch and redeployment strategies, they have not considered the existence of these classification errors. For example, Brotcorne et al. [10] and Jagtenberg et al. [11] revealed that the greedy policy of allocating the nearest ambulance to patients does not always yield the best performance. Moreover, research into optimizing decisions in real time has achieved more realistic results [12]. Maxwell et al. [13], Nasrollahzadeh et al. [14], Maxwell et al. [15], and Schmid [16] all showed that the approximate dynamic programming (ADP) model works well as a real-time ambulance model of operational policy optimization. However, although the ADP produced a near-optimal solution in limited experiments, all of these studies assumed one type of ambulance and no classification errors.

Thus, more sophisticated two-tiered ambulance operations are required that consider the existence of classification errors. Furthermore, it is important to determine (1) how the optimal operation policy changes according to the classification errors and (2) what type of classification decision should be taken for ambiguous patients to minimize patient risk. Some studies have considered the classification of patient severity in mixed ALS/BLS systems by categorizing patients into types based on their severity [6, 17, 18]. However, these studies all assumed that patient severity can be immediately and accurately determined when the call is received. Furthermore, few studies have considered the possibility of errors when classifying patient severity during ambulance operations. McLay and Mayorga [19] mathematically addressed patient classification errors during ambulance operations. They classified patient priorities in the all-ALS system into three levels and optimized the ambulance operation policy by using the Markov decision process (MDP) model. They then compared two cases, in which middle-priority patients were classified as high-risk and low-risk patients.

In this context, we propose an approximate dynamic programming (ADP) model that runs on a discrete event simulation to optimize the dispatch-and-redeployment policy of a two-tiered ambulance system by considering errors in patient-severity classification. The computational experiment environment was created based on actual historical data from Seoul by considering the probability distribution of demand-and-service time, time-varying demand, and traffic speed. The computational experiments show that our proposed algorithm performs better than the greedy policy. In addition, we identify the influence and correlation between classification errors and the ratio of ALS units to BLS units based on patient risk level. This can provide insights into patient-classification attitudes and ambulance management strategies.

## 2. Problem Description

In this study, we use an ADP algorithm to optimize ambulance dispatch and redeployment decisions in order to reduce the risk level of patients through rapid transportation. The approach assumes that the strategic level of decision-making, such as the location of the emergency center and hospital and the number of ambulances, is fixed. In addition, real-time dispatch and redeployment decisions are dealt with at the operational level. The ambulance operating environment is assumed to comprise a two-tiered ambulance, two types of patient classes with different severities, and patient classification errors. These considerations are not only key factors influencing decision-making but are also close to that of an actual ambulance operating environment.

Patients calling the emergency services are classified into two groups: high-and low-risk patients with high and low severity levels, respectively. We denote the severity of patients as *H*^A^ (*L*^A^) if the actual severity of the patient is high (low) risk, and *H*^C^ (*L*^C^) if the classified severity of the patient is high (low) risk. High-risk patients are described as life-threatened if they do not receive adequate treatment within a given response time threshold (RTT). Although low-risk patients are not life-threatened, it is preferable to treat them quickly to increase the service satisfaction level and prevent their treatment from becoming complicated and turning them into high-risk patients.

**Figure 1:**
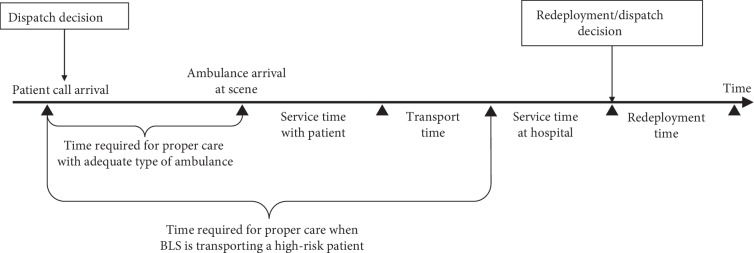
Flowchart of the ambulance operation process and decision-making points.

The operation process of the ambulance and the time spent during the process are shown in Figure 1. The ambulances typically remain at the emergency center. When a patient is reported, the decision maker decides which ambulance to send to the patient using information of the severity classification. When an ambulance arrives at the patient location, the actual severity of the patient becomes known, and the patient receives a first-aid service. The ambulance then transports the patient to the nearest hospital emergency room. After the ambulance arrives at the hospital, the patient is transferred to the hospital staff. After delivering the patient to the hospital, the decision maker determines whether there are any patients waiting to be allocated an ambulance. If such a patient exists, the ambulance is allocated to the patient; if a patient does not exist, the decision maker determines which emergency center the ambulance should be relocated to. When a patient is reported and no ambulance is available, which is a rare occurrence in reality and has thus far not been noted in any previous experiments, the patient is placed in a virtual queue. In this situation, when an ambulance is about to be placed into an idle state, a high-risk patient is allocated at a higher priority than low-risk patients, regardless of the report-arrival time. For patients within the same risk level, an ambulance is allocated on a first-come-first-served basis. If an ambulance is idle when a patient is waiting, the ambulance must respond to the patient, regardless of the location of the patient; i.e., a delay in ambulance allocation is not allowed.

The response time (RT), which is typically used as an evaluation measure of the EMS system, denotes the time from the patient report being obtained at the emergency center to the ambulance arriving at the scene. However, in this study, we use the time required for proper care (RT_PC), which is the time from the patient report being obtained at the emergency center to the patient beginning to receive appropriate treatment. That is, unless the ambulance is the correct type to handle the severity of the patient, the patient only begins receiving appropriate treatment once the ambulance arrives at the hospital. For example, if an ALS transports a high- or low-risk patient, or if a BLS transports a low-risk patient, the RT_PC does not differ from the original RT. However, when a BLS transports a high-risk patient, providing appropriate treatment quickly is complicated by the lack of specialized medical resources, such as a respirator or emergency medical staff [20]. Thus, the end time for the RT_PC is the time that the ambulance arrives at the hospital. The criterion for measuring RT_PC is also expressed in Figure 1.

**Figure 2:**
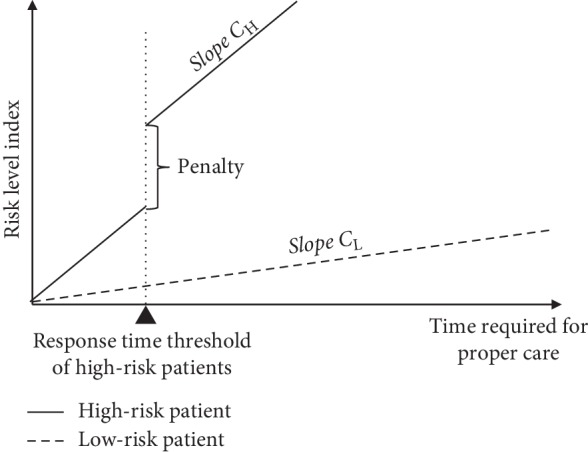
Risk level index function.

In this study, we propose a risk level index (RLI) that reflects the different risk levels of patient groups with different severity as another performance measure of the EMS system. RLI is the response time adjusted to the risk of the patient. The RLI function *f*(RT, *S*^A^) is a function of RT_PC and actual patient severity (*S*^A^), as shown in equation (1) and Figure 2:

(1) $$\begin{array}{c} f\left(\text{RT},S^{\text{A}}\right)=\left\{\begin{array}{cc} C_{\text{H}}\cdot \text{RT\_PC}+1\left\{RT\_PC>RTT\right\}\cdot \text{Penalty,} & \text{if }S^{\text{A}}=H^{\text{A}},\\ C_{\text{L}}\cdot \text{RT\_PC,} & \text{if }S^{\text{A}}=L^{\text{A}}. \end{array}\right. \end{array}$$

The RLI increases linearly with RT_PC but with different slopes depending on the severity of the patient. When the RT_PC of high-risk patients exceeds the RTT, a penalty of constant value is added. The value of these parameters can be set according to the decision of an EMS system manager if *C*_H_ ≥ *C*_L_ ≥ 0. The RTT is typically set to 8 min or 9 min [21, 22].

The evaluation index of ambulance operations in EMS systems usually includes the RT [16, 23], the survival rate, which is a continuous function of RT [24–28], and the coverage level, which is the proportion of reports covered within a predefined RTT [29, 30]. However, these have some limitations. First, it is difficult to use the RT index to consider the difference among each patient group with different severities and determine whether the report is covered within the RTT. Second, the quantitative measurement of survival rate over RT is not easily medically validated due to the different status levels of each patient; thus, previous studies used different survival-rate functions. In addition, higher priority might be assigned to a patient whose survival rate is high but rapidly decreasing than to a patient whose survival rate is already low; this raises an ethical issue. Lastly, as the coverage level only checks whether RT is within RTT, it does not evaluate the exact RT; this might cause the time immediately before the RTT to be labeled as the RT, neglecting the condition of “the sooner, the better” and potentially ignoring patients already waiting for longer than RTT. Conversely, the RLI used in this study has advantages of including all characteristics, such as the patient's severity, RT, and coverage level.

The proposed RLI is not an entirely new concept as several studies have used an objective function that either considers the risk associated with matching ambulance type and patient severity [6, 14] or that considers a linearly increasing risk over time with a penalty for exceeding the time threshold [31]. The RLI function for high-risk patients can be viewed not only as the response time adjusted by the patient risk but also as a weighted sum of multiple objectives, the RT and the coverage level, whereas the RLI function for low-risk patients is a relatively low-weighted RT.

When classifying patients as high or low risk, two types of error may occur (Table 1). The undertriage rate *α* is the probability of classifying a high-risk patient (*H*^A^) as a low-risk patient (*L*^C^), and the overtriage rate *β* is the probability of classifying a low-risk patient (*L*^A^) as a high-risk patient (*H*^C^). The purpose of this study is not to determine the exact value of these errors but to investigate the influence of these errors; thus, the authors assumed that errors *α* and *β* are known in advance by using historical data.

Moreover, the ratio of actual high-risk patients to all patients (Pr_H^A^_) is assumed to be known from the historical data. In this study, Pr_H^A^_ = 24.8%, according to a survey by Vandeventer et al. [32]. Therefore, if *α*, *β*, and Pr_H^A^_ are known, we can calculate the probability of a patient being correctly classified as high risk (Pr_H^A^|H^C^_) and vice versa (Pr_L^A^|L^C^_):

(2) $$\begin{array}{c} {\text{Pr}}_{{\text{H}}^{\text{A}}\left|{\text{H}}^{\text{C}}\right.}=\frac{\left(1-\alpha \right){\text{Pr}}_{{\text{H}}^{\text{A}}}}{\left(1-\alpha \right){\text{Pr}}_{{\text{H}}^{\text{A}}}+\beta \left(1-{\text{Pr}}_{{\text{H}}^{\text{A}}}\right)},\\ {\text{Pr}}_{{\text{L}}^{\text{A}}\left|{\text{L}}^{\text{C}}\right.}=\frac{\left(1-\beta \right)\left(1-{\text{Pr}}_{{\text{H}}^{\text{A}}}\right)}{\alpha \cdot {\text{Pr}}_{{\text{H}}^{\text{A}}}+\left(1-\beta \right)\left(1-{\text{Pr}}_{{\text{H}}^{\text{A}}}\right)}. \end{array}$$

## 3. Model and Solution Algorithm

The process of the EMS system is modeled as a semi-MDP model that runs on discrete event simulation. The state transition function depends partly on the controllable decisions of dispatch and redeployment and partly on unmanageable stochastic events, such as patient arrival and service completion. A decision is made at the time an event occurs that requires new decision-making. In other words, the simulation time jumps to the real time of the next event instead of adding a constant unit of time. Thus, multiple calls are never received simultaneously. Here, we let *τ*_*t*_ denote the time when the *t*^*th*^ event occurs.

In a semi-MDP environment, dynamic programming (DP) can be used to obtain optimal policies. DP uses the state *S*, action *a*, contribution function *C*(*S*, *a*), and transition probabilities. In this study, *S* represents the state of the EMS system associated with the ambulance and the patient. The state of ambulance *i* is denoted by vector *a*_*i*_={*a*1,  *a*2,   …,  *a*6}, and the state of patient *j* is denoted by vector *p*_*j*_={*p*1, *p*2,…, *p*4}. Attributes *a*1–*a*6 represent the ambulance type (ALS or BLS), ambulance location, ambulance status (idle, moving toward patient, service in patient's location, moving toward hospital, service in hospital, and moving back toward emergency center), patient ID if the ambulance is assigned to the patient, destination (specific patient/hospital/emergency center) if the ambulance is in transit, and time remaining until arrival at destination, respectively. Attributes *p*1–*p*4 represent the patient's location, time when an incident is reported, status (waiting/in service), and classified severity, respectively. The set of all ambulances is *𝒜*, and the set of all patients is *𝒫*. The state *S*_*t*_ of event *t* at time *τ*_*t*_ is represented as a vector {*a*_*i*_, *p*_*j*_}_*i*∈*𝒜*,*j*∈*𝒫*_. Action *a* decides which idle ambulance to send to which waiting patient, or which emergency center to relocate an ambulance to that has just been labeled idle after completing its service to a hospital.

The contribution function *C*(*S*_*t*_, *a*_*t*_) returns the value of the reward given when action *a*_*t*_ is performed in state *S*_*t*_. In this study, we define the expected RLI of the patient as the reward value, and the contribution function is described in equation (3). As the exact RT is not known when performing action *a*_*t*_ at state *S*_*t*_, the average RT for the distance is used:

(3) $$\begin{array}{c} C\left(S_{t},a_{t}\right)=\left\{\begin{array}{cc} {\text{Pr}}_{{\text{H}}^{\text{A}}\left|{\text{H}}^{\text{C}}\right.}\cdot f\left(\text{RT},H^{\text{A}}\right)+{\text{Pr}}_{{\text{L}}^{\text{A}}\left|{\text{H}}^{\text{C}}\right.}\cdot f\left(\text{RT},L^{\text{A}}\right), & \text{if ALS carry }H^{\text{C}}\text{ patient,}\\ {\text{Pr}}_{{\text{H}}^{\text{A}}\left|{\text{L}}^{\text{C}}\right.}\cdot f\left(\text{RT},H^{\text{A}}\right)+{\text{Pr}}_{{\text{L}}^{\text{A}}\left|{\text{L}}^{\text{C}}\right.}\cdot f\left(\text{RT},L^{\text{A}}\right), & \text{if ALS carry }L^{\text{C}}\text{ patient,}\\ {\text{Pr}}_{{\text{H}}^{\text{A}}\left|{\text{H}}^{\text{C}}\right.}\cdot f\left(\text{RT},H^{\text{A}}\right)+{\text{Pr}}_{{\text{L}}^{\text{A}}\left|{\text{H}}^{\text{C}}\right.}\cdot f\left(\text{RT},L^{\text{A}}\right), & \text{if BLS carry }H^{\text{C}}\text{ patient,}\\ {\text{Pr}}_{{\text{H}}^{\text{A}}\left|{\text{L}}^{\text{C}}\right.}\cdot f\left(\text{RT},H^{\text{A}}\right)+{\text{Pr}}_{{\text{L}}^{\text{A}}\left|{\text{L}}^{\text{C}}\right.}\cdot f\left(\text{RT},L^{\text{A}}\right), & \text{if BLS carry }L^{\text{C}}\text{ patient.} \end{array}\right. \end{array}$$

*X* ^*π*^(*S*_*t*_) is a function that returns the action to be taken in state *S*_*t*_, when policy *π* is used. The greedy policy *π* minimizes *C*(*S*_*t*_,  *X*^*π*^(*S*_*t*_)); that is, at every decision point, an action is taken by only considering the reward that can be received at the current state. However, we aim to obtain a policy that considers the effects of the current action on future situations. Thus, ADP is used to find a policy that minimizes the expected value of the total discounted sum of the patient's RLI over a long period, ∑_*t*=0_*γ*^*t*^*C*(*S*_*t*_, *X*^*π*^(*S*_*t*_)), where *γ* ∈ [0,1] is a discount factor expressing how much future rewards are worth in the present. As the time interval between rewards is not constant and cannot be precisely predicted in advance, the discount rate is set to a constant for simple application. *V*(*S*_*t*_) denotes the value of being in state *S*_*t*_ under policy *π*; that is, the expected value of the total discounted sum of the RLI. Then, *V*(*S*_*t*_) can be recursively expressed using the Bellman equation form, as in equation (4). *W*_*t*_ denotes the external information related to the status change known between *τ*_*t*−1_ and *τ*_*t*_; for example, obtaining a new patient call and the ambulance arrival time at the patient location or hospital. Let the state transition function be *S*^*M*^, then *S*^*M*^(*S*_*t*_, *a*_*t*_, *W*_*t*+1_) represents the state at time *τ*_*t*+1_when external information *W*_*t*+1_ is received after taking action *a*_*t*_ in state *S*_*t*_, which is *S*_*t*+1_:

(4) $$\begin{array}{c} V\left(S_{t}\right)=\underset{a_{t}}{\min}\left(C\left(S_{t},a_{t}\right)+\gamma Ε\left[V\left(S^{M}\left(S_{t},a_{t},W_{t+1}\right)\right)\right]\right). \end{array}$$

The size of the state space increases rapidly as the problem size becomes larger; i.e., as the dimensions of state *S* and external information *W* increases. Thus, the calculation of every *V*(*S*_*t*_) in the reverse direction starting from *V*(*S*_*T*_) at terminal time *τ*_*T*_ within a reasonable time is almost impossible as *V*(*S*_*t*_) is evaluated for all states *S*_*t*_ ∈ *𝒮*. Therefore, we used ADP, which is a type of reinforcement learning and a powerful tool for solving stochastic and dynamic problems and making real-time decisions [33]. ADP approximates *V*(*S*_*t*_) iteratively and in the forward direction. It makes a decision to minimize $\hat{v}$ at each iteration and decision point. In equation (5), $\hat{v}$ is a sample estimate of the value of being in state *S*_*t*_^*n*^ obtained in iteration *n* at time *τ*_*t*_, and $\bar{V}$ is a value function that returns an approximate value of being in a certain state obtained from all previous steps:

(5) $$\begin{array}{c} \hat{v}=\underset{a_{t}}{\min}\left(C\left(S_{t}^{n},a_{t}\right)+\gamma Ε\left[\bar{V}\left(S^{M}\left(S_{t}^{n},a_{t},W_{t+1}\right)\right)\right]\right). \end{array}$$

$\hat{v}$ is used to update $\bar{V}\left(S_{t}^{n}\right)$ to make it more accurate, as shown in equation (6), and *δ*_*s*_^*n*,*t*^ is the step size in iteration *n* at time *τ*_*t*_, where 0 ≤ *δ*_*s*_^*n*,*t*^ ≤ 1.

(6) $$\begin{array}{c} \bar{V}\left(S_{t}^{n}\right)⟵\left(1-\delta_{s}^{n,t}\right)\bar{V}\left(S_{t}^{n}\right)+\delta_{s}^{n,t}{\hat{v}}_{t}^{n}. \end{array}$$

**Figure 3:**
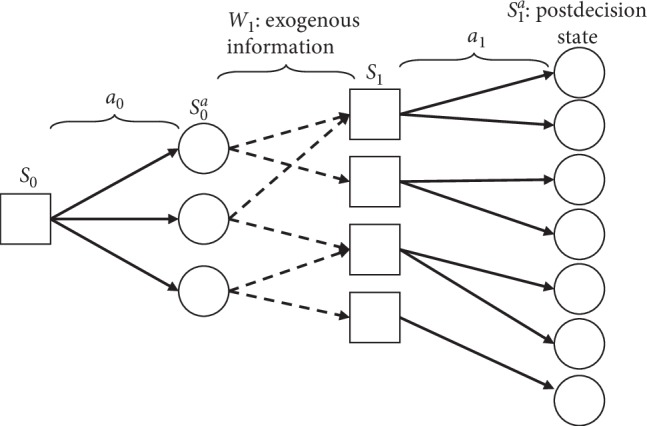
State-transition diagram.

The ADP further uses the postdecision state and aggregation techniques to increase the computation speed. Postdecision state *S*_*t*_^*a*^ represents the state immediately after the decision to take action *a* at time *τ*_*t*_ and before the external information *W*_*t*+1_ is received. Thus, after a decision is made to perform action *a*_*t*_ in state *S*_*t*_, as shown in Figure 3, the time does not elapse and the process goes into postdecision state *S*_*t*_^*a*^ deterministically. Next, external information is received between *τ*_*t*_ and *τ*_*t*+1_, and the process goes into state *S*_*t*+1_. The ADP at the current time *τ*_*t*_ estimates the value of postdecision state *S*_*t*_^*a*^ instead of state *S*_*t*+1_ by using equation (7) instead of equation (5); thus, calculation of the expectation value in equation (5) can be omitted. The ADP has a large computational advantage for estimating the value of being in a postdecision state, as it can use the deterministic value of postdecision state at the decision point instead of computing possibilities of reaching the next state *S*_*t*+1_ for all possible states:

(7) $$\begin{array}{c} \hat{v}=\underset{a_{t}}{\min}\left(C\left(S_{t}^{n},a_{t}\right)+\gamma \bar{V}\left(S_{t}^{a,n}\right)\right). \end{array}$$

Furthermore, $\bar{V}$ is now a value function that returns an approximate value of being in postdecision state *S*_*t*_^*a*,*n*^ and is updated using equation (8) instead of equation (6):

(8) $$\begin{array}{c} \bar{V}\left(S_{t}^{a,n}\right)⟵\left(1-\delta_{s}^{n,t}\right)\bar{V}\left(S_{t}^{a,n}\right)+\delta_{s}^{n,t}\hat{v}. \end{array}$$

In addition, aggregation is used to reduce computation and generalize the evaluation of the value function across other similar states. Different but similar states are aggregated only to approximate the value function at the decision-making point. After the decision, the states are disaggregated and proceed to the next simulation event. In this study, temporal and spatial aggregations are used. The temporal and spatial aggregation sets are, respectively, denoted as *ϕ*^TA^ and *ϕ*^SA^, where the levels are |*ϕ*^TA^|=3 and |*ϕ*^SA^|=9. Temporal aggregation is achieved by dividing the day into three time zones as 01 : 00–08 : 00 (*ϕ*_1_^TA^), 08 : 00–11 : 00 (*ϕ*_2_^TA^), and 11 : 00–01 : 00 (*ϕ*_3_^TA^), depending on the incidents; each of these time zones has similar demands (calls). For the spatial aggregation, the space is divided into a grid divided into nine squares with three equal sections along both the horizontal and vertical axes. The state's attributes used for the evaluation are the number of idle or relocating ambulances and the number of patients waiting to be allocated an ambulance. Other attributes of the state are omitted.

In other words, the aggregated state that stores the value of the value function is a vector of 19 dimensions consisting of the number of idle ambulances and pending patients in each of nine square regions and the time zone. The value of the value function for all aggregated states is stored in a lookup table. Aggregation reduces the size of the table, and the use of the postdecision state reduces the number of times a table is queried. Algorithm development process in this study so far builds on previous research into the ambulance dispatch and redeployment problem by additionally considering multiple types of patients and ambulances, and patient classiﬁcation errors; so, we recommend to see [16, 33] for full details.

However, as it is still a large table, we also use the monotonicity-preserving projection operator Π_M_ introduced by Jiang and Powell [34]. If the expected contribution between some states can be compared in advance, this operator can be used to reduce the computation by efficiently approximating the value function. In this study, if state *S* has (1) a greater or equal number of idle ALS vehicles at each emergency center, (2) a greater or equal number of idle ambulances at each emergency center, (3) a lesser or equal number of pending high-risk patients in each region (aggregated space), and (4) fewer patients who have not been assigned an ambulance in each region than state *S*′, then being in state *S* would result in better contributions than being in state *S*′. If *S* dominates *S*′ as described, *S≽S*′. In this study, because the aim is to minimize RLI, *V*(*S*), the expected value of being in state *S*, should be less than *V*(*S*′) if *S≽S*′.

Let *s*^*r*^ ∈ *𝒮* be a reference state, *z*^*r*^ ∈ *ℝ* be a reference value, and (*s*^*r*^, *z*^*r*^) be a reference point for comparison. The value function is *V* ∈ *ℝ*^d^, and the monotonicity-preserving projection operation is defined as Π_M_ : *𝒮* × *ℝ* × *ℝ*^d^⟶*ℝ*^d^. The component of the output vector of Π_M_ at state *s* is defined as

(9) $$\begin{array}{c} \Pi_{M}\left(s^{r},z^{r},V_{t}\right)\left(s\right)=\left\{\begin{array}{cc} z^{r}, & \text{if }s=s^{r},\\ z^{r}\wedge V_{t}\left(s\right), & \text{if }s^{r}≼s,\;s\neq s^{r},\\ z^{r}\vee V_{t}\left(s\right), & \text{if }s^{r}≽s,\;s\neq s^{r},\\ V_{t}\left(s\right), & \text{otherwise.} \end{array}\right. \end{array}$$

In general, for every iteration of ADP, the Π_M_ operator is applied every time after updating the value function with value *z*^*r*^ for current state *s*^*r*^. Jiang and Powell [34] showed that the value function converges quickly with fewer iterations because the monotonicity of the state set is always maintained by using the Π_M_ operator as follows:

(10) $$\begin{array}{c} \bar{V}⟵\Pi_{M}\left(s^{r},z^{r},\bar{V}\right). \end{array}$$

However, if the Π_M_ operator is used at every decision-making instant, the time required per iteration is greatly increased, although the number of iterations is reduced because the reference state is compared with all other states. Therefore, in this study, the Π_M_ operator is applied stochastically to take advantage of the computational time. At the end of each iteration, we probabilistically sample ten states for each time zone, with the probability being proportional to the number of visits to that state. Then, with only the sampled states as reference points, all other states are updated through the Π_M_ operator. Using the stochastic monotonicity-preserving projection, the approximation of the value function can be effectively updated by applying the Π_M_ operator in a much more time-efficient manner.

**Figure 4:**
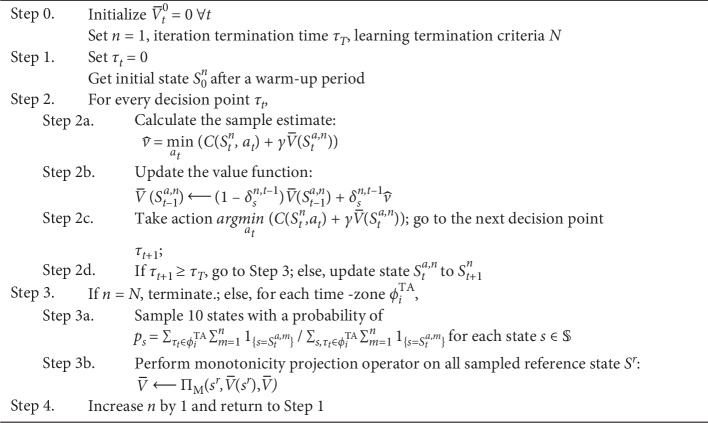
Details of the mini-batch monotone-ADP algorithm.

Here, we propose the mini-batch monotone-ADP algorithm, which stochastically uses the monotonicity-preserving projection to modify the monotone-ADP algorithm proposed in the study by Powell [33]. The detailed algorithm is shown in Figure 4. The initial value of $\bar{V}$ affects the tradeoff relationship between exploration and exploitation. In this study, as the minimization problem is considered, the initial value of $\bar{V}$ is set to 0 to explore as many action decisions as possible.

## 4. Numerical Experiments and Results

### 4.1. Experimental Design

**Figure 5:**
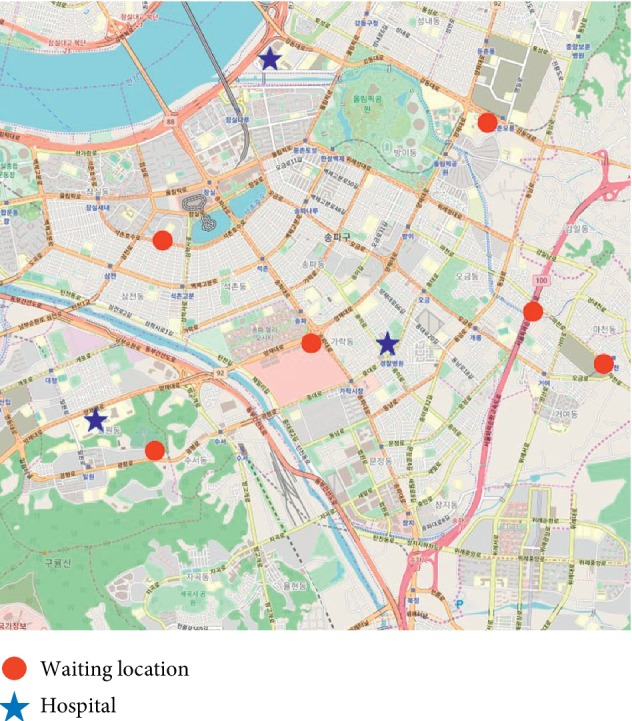
Locations of emergency centers and hospitals in Songpa-gu, Seoul.

**Figure 6:**
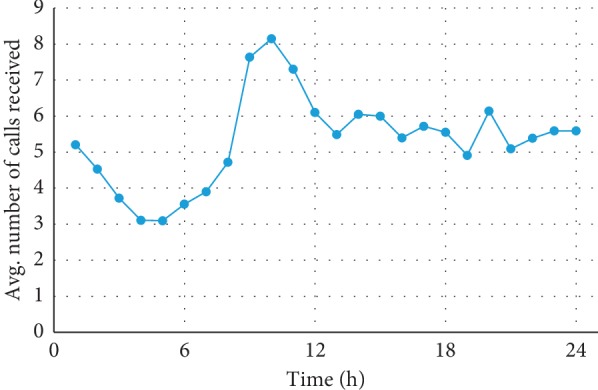
Average arrival time of all emergency calls received in one day.

**Figure 7:**
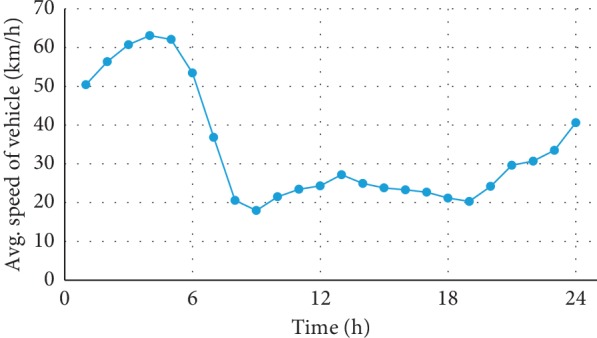
Average vehicle speeds over a day.

This study used actual data obtained on March 2015 for Songpa-gu, Seoul, Korea. Songpa-gu is a high-density neighborhood with a population of approximately 680,000 and an area of approximately 90 km^2^. Actual historical data on patient arrival rate and traffic was obtained from the South Korean Open Data Portal (data.go.kr) and the Seoul Traffic Information Center, respectively. These data reveal an average of 127.9 calls per day, of which 24.8% are assumed to be high risk [32] The area contains three hospitals with emergency rooms, six ambulances, and six fire stations that function as waiting locations (Figure 5). Actual data on the time-varying demand and changes in ambulance speed over time were also used in the model. Patient calls were generated from a Poisson process with different parameters for each district, and the arrival time of the calls at each district was also generated using a Poisson process with a time-varying parameter. The average number of patients who arrived from the entire Songpa-gu area over time is shown in Figure 6, and the average speed of an ambulance in traffic is shown in Figure 7.

It is assumed that up to two ambulances can be placed in a waiting location at one time. The coefficients of the RLI function were set to *C*_H_=1, *C*_L_=0.25, Penalty=30, and RTT=7 min, respectively, based on basic interviews with EMS practitioners. As the RLI can be viewed as an adjusted RT, this setting means that exceeding the RTT is equivalent to a 30-min delay. Moreover, 4 min for a high-risk patient is equal to 1 min for a low-risk patient. However, different values can be applied depending on the practitioners' opinion. The service time at the patient's location was assumed to follow a gamma distribution with a scale parameter *θ* = 3.57 and a shape parameter *k* = 6.2, with an average of 22.12 min. The service time at the hospitals was also assumed to follow a gamma distribution with a scale parameter *θ* = 5.02 and a shape parameter *k* = 3.0 with an average of 15.05 min, as inferred by Maxwell et al. [15].

The step size of the proposed ADP algorithm was set to *δ*_*s*_^*n*,*t*^=1/∑_*t*_∑_*m*=1_^*n*^1_{*s*=*S*_*t*_^*a*,*m*^}_, which is the reciprocal of the number of visiting states *S* from the beginning to time *τ*_*t*_ at iteration *n*. This step size almost definitely assures convergence as the number of iterations increases with a well-known result in stochastic approximation because it satisfies ∑_*n*=0_^*∞*^*δ*_*s*_^*n*,*t*^=*∞* and ∑_*n*=0_^*∞*^(*δ*_*s*_^*n*,*t*^)^2^ < *∞*, with some regularity assumptions regarding the underlying stochastic processes (see the studies by Jiang and Powell [34] and Ryzhov et al. [35] for details). The discount factor *γ* was set to 0.9, which is future-oriented and showed the best performance in simple tests. The algorithm was implemented in Python, and all experiments were run on a computer with an i5-4460 CPU. We varied the following three factors to determine their influence on the RLI: the ratio of ALS to the total number of ambulances (hereafter the ALS ratio), the undertriage rate *α*, and the overtriage rate *β*. In this experiment, the ALS ratios with respect to the six ambulances were 0.0, 0.17, 0.33, 0.5, 0.67, 0.83, and 1.0. Each error *α* and *β* was divided into five increments of 0.1, beginning at 0. A complete factorial experiment was performed for each combination of factors.

**Figure 8:**
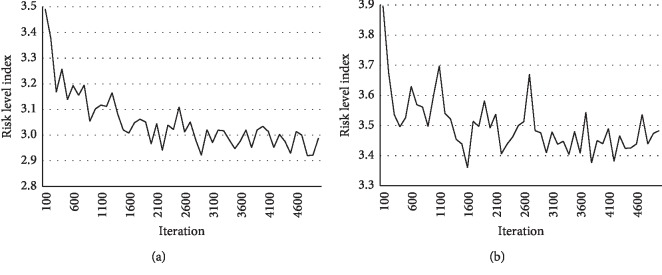
Risk level index for iterations of the learning phase: (a) *α*=*β*=0, ALS Ratio=0.83 and (b) *α*=*β*=0.4, ALS Ratio=0.83.

The learning phase of the proposed ADP algorithm, which approximates the optimal value function, was terminated based on a two-*h* limit instead of the number of iterations. We drew each point in Figure 8 to represent the average value of the RLI for 100 iterations. As Figure 8 shows, the RLI gradually decreased and converged after an average of 5387.7 iterations. The policy optimized by the proposed ADP algorithm (hereafter the ADP policy) was tested 100 times for each experiment. Each iteration of the learning phase and each test of optimized policy were run for seven simulation days after a warm-up time of one simulation day, which is sufficient time to eliminate the influence of an arbitrary initial position of the ambulances.

### 4.2. Comparison with Greedy Policy

As mentioned in Section 3, the greedy policy moves the ambulance in a way that minimizes *C*(*S*_*t*_,  *X*^*π*^(*S*_*t*_)); thus, it only considers the contribution at the current state and does not consider the effects of the current action on future situations. However, because it still considers the patient severity classification errors, ambulance type, and the expected response time of the current state, it is a basic and reasonable policy that is expected to perform at least better than a myopic policy that allocates the nearest ambulance to the patient and relocates the former to the nearest available waiting location.

It is difficult to compare the various policies with a small number of simulation experiments because the RLI has high variability due to the inherent uncertain nature of patient numbers in the EMS system. Therefore, we used the common random number (CRN), a variance reduction technique, to efficiently compare alternative policies with a small number of simulations. This was made possible because the CRN method synchronizes a random number stream for some variables to generate the same random number in every alternative policy when running the simulation. In this study, we compared the greedy policy and the ADP policy under the condition that the random number streams of the patients' occurrence times, locations, and actual and classified severities were synchronized.

**Figure 9:**
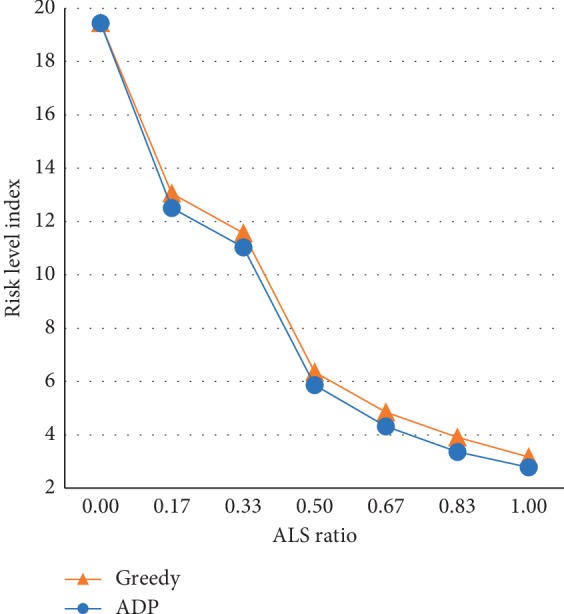
Comparison of the risk level index for greedy and ADP patient transport policies based on the ALS (advanced life support) ratio.

To find the minimum number of ALS vehicles capable of effectively transporting high-risk patients, the average RLI according to the ALS ratio was analyzed as shown in Figure 9. Figure 9 shows that the RLI increased sharply when the ALS ratio decreased to less than 0.5. This was a result of the frequent assignment of BLS to high-risk patients because there were insufficient ALSs to treat them. In a further experiment that restricted BLS from transporting high-risk patients, these patients continued to accumulate in the queue if the ALS ratio was less than 0.5. This indicates that the ALS in the EMS system had insufficient capacity; therefore, subsequent analyses of the experimental results will only evaluate situations in which the ALS ratio is above 0.5.

Table 2 shows the results of the RLI of the two policies for each of the four ALS ratios and five levels of *α* and *β*. In the paired *t*-test for the ADP and greedy policies, the former performed significantly better in 97 of 100 combinations at the 95% confidence level. In most experiments, the *p* value was less than 0.001, indicating that the dominant performance was very significant.  Table 3 shows the difference in RLI between the ADP and the greedy policy based on the ALS ratio, which had the greatest effect on patient risk level. Overall, the patient RLI decreased by 0.486 when using the ADP policy, which was an improvement of 11.2% over the greedy policy.

### 4.3. Factors Affecting the Risk Level Index

**Figure 10:**
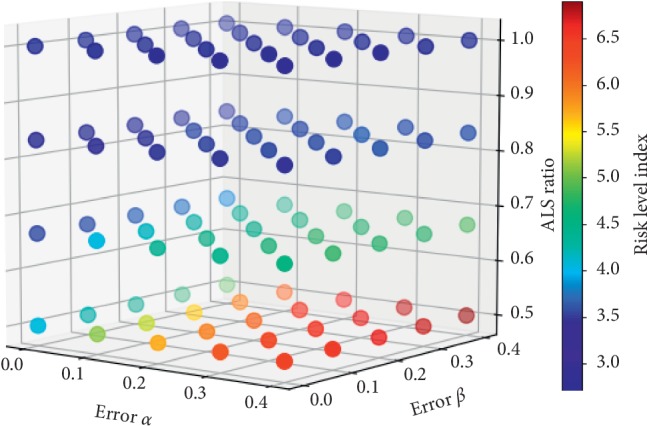
Risk level index plot for the ALS ratio, error *α*, and error *β*.

The results of multiway ANOVA tests on the RLI in the ADP policy are shown in Table 4 and Figure 10. The ANOVA was conducted using SAS software. As expected, the RLI decreased with increasing ALS ratio and decreasing undertriage rate *α* or overtriage rate *β*. The main effects on error *α*, error *β*, and ALS ratio were significant, as were the interaction effects of the *α* × ALS ratio and *β* × ALS ratio, with a significance level of 0.01 and a *p* value of less than 0.001. The interaction effect of *α* × *β* was significant with a *p* value of less than 0.05, but the magnitude of the effect was negligible; therefore, a detailed analysis was not conducted. The interaction effect of *α* × *β* × ALS ratio was not significant. The ALS ratio had the greatest impact on the RLI, followed by *α*, the *α* × ALS ratio interaction, the *β* × ALS ratio interaction, and *β*. Figure 10 shows that each factor has a nonlinear effect on RLI.

**Figure 11:**
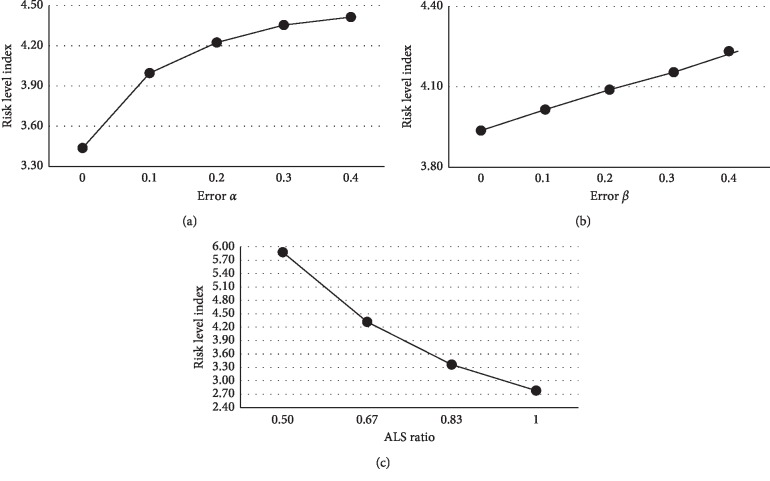
Effect of (a) error *α*, (b) error *β*, and (c) ALS ratio on the patient risk level index.

**Figure 12:**
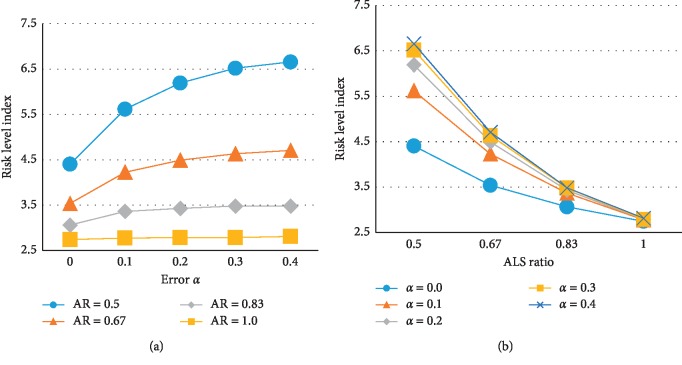
Interaction effect of ALS ratio and error *α* on the risk level index.

**Figure 13:**
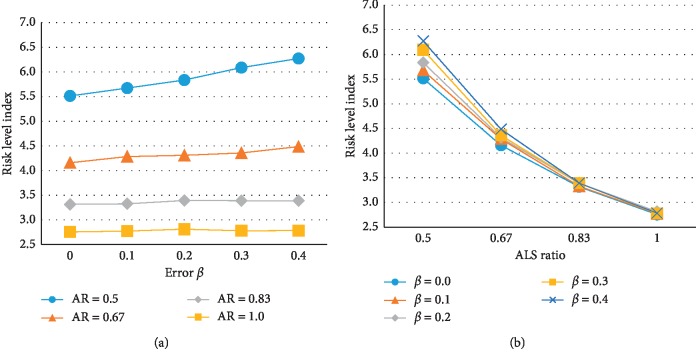
Interaction effect of ALS ratio and error *β* on the risk level index.

The main effects of the different factors are summarized in Figure 11 using the averages of the experimental values for all levels of the factors. The effect of undertriage rate was distinctly nonlinear; RLI increased rapidly as *α* increased from 0 to 0.1. Moreover, when error *α* increased, there was an increased frequency of assigning a BLS to misclassified actual high-risk patients, leading to a negative impact on the patient's risk level. Conversely, the RLI increased linearly with increasing *β*; however, this effect was not large because assigning ALS to a misclassified actual low-risk patient does not immediately and directly increase that patient's risk level, but rather indirectly affects the ability of future high-risk patients to cope. Another reason for the small effect is that the absolute number of high-risk patients is relatively small. As the ALS ratio decreased, the RLI increased more rapidly. Figures 12 and 13 show the interaction effect between the undertriage rate *α* or overtriage rate *β* and ALS ratio on the RLI. As the ALS ratio increased, the RLI was less affected by both classification errors; however, when the ALS ratio was relatively low, *α* generated a greater difference in RLI than *β*.

### 4.4. Operational Properties of the Improved Ambulance Operation Policy

Although the ADP policy performs better than the greedy policy, understanding how ambulance operations based on the ADP policy differ from those of the greedy policy is complex. Thus, to gain a greater understanding of operational properties and more general and intuitive insights into decision-making in the proposed optimized ambulance operation policy, we developed and analyzed additional indices other than RLI.

The first of these indices, the future orientation for patients classified as high-risk index (FHI), refers to the ratio achieved when the nearest ALS is not allocated, or the nearest ambulance is not allocated to a patient classified as high risk when other idle ALSs are present. The FHI was close to 0 in almost all situations (Table 5). As the availability of an ALS increased as the ALS ratio increased, the FHI increased slightly from 0.05 to 0.09, which was slightly future-oriented but still very low. This means that almost all patients classified as high risk, regardless of the magnitude of the error and the ALS ratio, were assigned the nearest ALS or a BLS if it was closer. In other words, ambulances tried to respond as quickly as possible to patients classified as high risk in any situation. On the contrary, dispatching ambulances to patients classified as low risk was less affected by the distance between the patient and the ambulance. On average, 30% of patients classified as low risk were assigned an ambulance other than the nearest ambulance.

The second index measured was the present orientation for patients classified as low-risk index (PLI), which refers to the ratio achieved when the ALS nearest to a low-risk patient is allocated when the nearest ambulance is that specific ALS and other idle ambulances are present. As the PLI value increased when a low-risk patient was assigned to the nearest ALS, a larger PLI value can be considered a more short-sighted dispatch approach, whereas a smaller PLI value is a more forward-looking dispatch approach. As a result, the PLI was minimally affected by the overtriage rate *β* (Table 6) but increased with increasing undertriage rate *α*. Thus, when the undertriage rate *α* was low, a patient classified as low risk was relatively frequently allocated an ambulance that is farther away, even if there was a closer ALS, in order to prepare for potential high-risk patients in future. On the contrary, when the undertriage rate *α* was high, the nearest ALS was frequently assigned to a patient even if they were classified as low risk. Furthermore, PLI exhibited nonlinear characteristics with undertriage rate *α*. When *α* increased from 0, the PLI increased considerably; however, when *α* exceeded 0.2, the increase in PLI was reduced.

Transporting a low-risk patient via ALS instead of BLS is a relatively inefficient way of using ambulance resources because it is an oversupply of the medical service. Thus, the third index measured was the inefficiency of the ALS index (IAI), which refers to the ratio achieved by allocating an ALS to a patient classified as low risk.  Table 7 shows the IAI for error *α* and error *β*, which is the average value of all experiments except for an ALS ratio of 1.0. IAI increased as *α* increased but was not significantly affected by *β*. In other words, as the undertriage rate increased, the inefficient use of ALS vehicles increased as more ALSs were assigned to patients classified as low risk. IAI also increased considerably and nonlinearly with *α*, similar to PLI. However, if the undertriage rate exceeded 0.2, the increase in IAI began to decrease. Finally, the average time required to relocate an ambulance was 2.61 min for the greedy policy and 3.84 min for the ADP policy. This indicates that although the greedy policy tried to relocate ambulances to make them idle as quickly as possible, the ADP policy tried to relocate ambulances to positions in which they could better respond to future patients, which led to improved performance.

## 5. Discussion

One of the major difficulties of an EMS system that transports patients by emergency ambulances is that they have to respond as quickly as possible despite limited ambulance resources. As not all patients are actual emergency patients, it is clear that using a mixed ALS/BLS system based on the severity of the patient's condition is a more efficient management strategy that will enable ambulances to respond to patients more rapidly. However, a key limitation of mixed ALS/BLS systems is the high risk of errors when classifying the severity of the patient's conditions.

Therefore, we developed an ADP model to optimize the ambulance dispatch and redeployment policy whilst including patient severity classification errors, which has not been sufficiently addressed by previous research. The patients were categorized into two groups: high risk (emergency) and low risk (nonemergency), where the majority fall into the latter category. A mixed ALS/BLS (two-tiered ambulance) system in which ALS and BLS vehicles are suitable for transporting high-risk and low-risk patients, respectively, was also considered. Two types of classification errors were assumed. The undertriage rate *α* was the probability of false classifications of actual high-risk patients, and the overtriage rate *β* was the probability of false classifications of actual low-risk patients. To develop a realistic model, system dynamics such as the time-varying traffic and frequency of patient occurrence and ambulance service time were based on historical data. As a result, the proposed ADP model reduced the risk level index (RLI) for all patients by an average of 11.2% compared to the greedy policy.

We also analyzed the magnitude and correlation of the effects of *α*, *β*, and the ALS ratio on the patient RLI under optimized ambulance dispatch and relocation policies. The patient RLI decreases when the ALS ratio increases or either classification error decreases. ALS ratio has the greatest impact on RLI, followed by *α*, *α* × ALS ratio interaction, *β* × ALS ratio interaction, and *β*. The interaction effects show that the patient RLI is less affected by changes in both classification errors as ALS ratio increases. Furthermore, a key observation is that *α* is much more sensitive than *β* in terms of the patient RLI. Therefore, it is desirable to classify patient severity in order to minimize the undertriage rate, even though it may increase the overtriage rate. For example, a patient whose condition is unclear or ambiguous and cannot be classified accurately would be classified as high risk. Furthermore, we evaluated the characteristics of the optimized ambulance operation policy. Patients classified as high risk were almost always assigned the nearest ALS regardless of the error level or ALS ratio. However, patients classified as low risk were more likely to be allocated the nearest ALS as the undertriage rate increased. Moreover, the manner in which ambulances operated was not significantly affected by the overtriage rate. These findings could serve as useful guidelines for optimizing ambulance operations when patient severity classification errors exist. Although the experimental environment was limited to Seoul, we expect that these results would not be significantly different in other regions with characteristics similar to Seoul, e.g., urban areas with a similar density of ambulance, base, and demand. However, in order to find out the impact of specific regional characteristics on the ADP policy, further research is required, such as learning a new policy in the area with different characteristics, e.g., rural areas with fewer ambulances and demands, or applying transfer learning using a policy that has completed learning in a similar area.

The main goal of this study was to develop an algorithm to determine the optimal ambulance operation policy using a realistic model that includes patient severity classification errors and then provide insights into classifying patient severity and ambulance operational strategy by identifying the effects of several factors and useful indices under the optimized policy. Therefore, the goal was not to demonstrate the superiority of an all-ALS system versus a mixed-ALS/BLS system or to increase the accuracy of patient classification. Although the total number of ambulances is assumed to be constant in this study, the number of ambulances available under one budget can vary due to differences in operating and purchasing costs between ALS and BLS. Increasing the total number of ambulances with a high ratio of BLS may contribute positively to reducing the patient risk level. In addition, if it was possible to lower the undertriage and overtriage rates, EMS system managers could consider controlling these errors and the configuration of ambulances to enable effective decision-making that could minimize the patient risk level within a limited budget. The findings of this study could be useful for such future research.

## Figures and Tables

**Table 1:** Probability of classification errors for patient severity.

Probability		Classified severity	
		High risk (*H*^C^)	Low risk (*L*^C^)
Actual severity	High risk (*H*^A^)	1 − *α*	*α*
	Low risk (*L*^A^)	*Β*	1 − *β*

**Table 2:** Risk level index of each ambulance operation policy, ALS ratio, undertriage rate *α*, and overtriage rate *β*.

ALS ratio	Undertriage rate *α*	Overtriage rate *β*									
		0		0.1		0.2		0.3		0.4	
		Greedy	ADP	Greedy	ADP	Greedy	ADP	Greedy	ADP	Greedy	ADP
0.5	0	5.12	3.99	5.26	4.21	5.48	4.32	5.57	4.61	5.68	4.89
	0.1	5.58	5.11	5.81	5.31	5.91	5.60	6.12	5.93^*∗*^	6.19	6.12^*∗∗*^
	0.2	5.98	5.75^*∗*^	6.34	5.97	6.30	6.12^*∗*^	6.49	6.45^*∗∗*^	6.62	6.67^*∗∗*^
	0.3	6.50	6.26^*∗*^	6.74	6.38	6.73	6.47^*∗*^	6.97	6.69	7.10	6.79
	0.4	7.00	6.45	7.19	6.49	7.41	6.68	7.46	6.78	7.52	6.90
0.67	0	4.07	3.43	4.13	3.45	4.18	3.52	4.20	3.59	4.30	3.70
	0.1	4.47	3.99	4.47	4.14	4.51	4.23	4.60	4.32	4.61	4.46^*∗*^
	0.2	4.78	4.38	4.77	4.47	4.89	4.40	4.90	4.52	4.93	4.68
	0.3	5.12	4.45	5.06	4.60	5.24	4.72	5.16	4.65	5.32	4.75
	0.4	5.43	4.55	5.49	4.75	5.48	4.70	5.69	4.69	5.59	4.84
0.83	0	3.47	2.99	3.47	3.03	3.57	3.09	3.55	3.12	3.58	3.09
	0.1	3.66	3.31	3.78	3.29	3.77	3.41	3.77	3.38	3.79	3.43
	0.2	3.90	3.39	3.88	3.40	3.90	3.45	3.89	3.43	3.95	3.47
	0.3	4.03	3.43	4.08	3.47	4.04	3.47	4.08	3.56	4.08	3.46
	0.4	4.17	3.46	4.23	3.45	4.17	3.54	4.30	3.46	4.36	3.49
1	0	3.18	2.71	3.17	2.72	3.19	2.8	3.15	2.76	3.16	2.73
	0.1	3.18	2.74	3.20	2.79	3.17	2.8	3.17	2.77	3.18	2.80
	0.2	3.18	2.74	3.18	2.76	3.17	2.83	3.17	2.77	3.17	2.83
	0.3	3.18	2.78	3.17	2.80	3.17	2.80	3.16	2.78	3.17	2.78
	0.4	3.14	2.81	3.16	2.80	3.16	2.85	3.17	2.82	3.16	2.79

Note. *p* is less than 0.001 in all experiments except ^*∗*^*p* < 0.05; ^*∗∗*^not significant.

**Table 3:** Difference in the risk level index between the ADP and greedy policies.

ALS ratio	Risk level index (ADP-greedy)		
	Average	Maximum	Minimum
0.5	−0.485 (8.0%)	−1.160 (22.0%)	0.000 (0.0%)
0.67	−0.535 (11.0%)	−0.996 (17.5%)	−0.156 (3.4%)
0.83	−0.536 (13.6%)	−0.872 (20.0%)	−0.346 (9.5%)
1	−0.389 (12.3%)	−0.467 (14.7%)	−0.314 (9.9%)
Average	−0.486 (11.2%)		

**Table 4:** ANOVA results of the risk level index.

Dependent variable: risk level index (RLI)					
Source	DF	Sum of squares	Mean square	*F* value	Pr > *F*

Model	99	16158.86	163.22	402.25	<.0001
Error	9900	4017.07	0.41		
Corrected total	9999	20175.93			

*R*-square	Coeff var	Root MSE	RLI mean		
0.801	15.592	0.637	4.085		

Source	DF	Anova SS	Mean square	*F* value	Pr > *F*
ALS ratio	3	13722.50	4574.17	11272.90	<.0001
*α*	4	1257.75	314.44	774.93	<.0001
*β*	4	107.24	26.81	66.07	<.0001
ALS ratio × *α*	12	933.14	77.76	191.64	<.0001
ALS ratio × *β*	12	112.73	9.39	23.15	<.0001
*α* × *β*	16	10.95	0.68	1.69	0.042
ALS ratio × *α* × *β*	48	14.56	0.30	0.75	0.9013

**Table 5:** Future orientation for patients classified as high-risk index (FHI) according to the ALS ratio.

ALS ratio	0.50	0.67	0.83	1

FHI	0.05	0.07	0.08	0.09

**Table 6:** Present orientation for patients classified as low-risk index (PLI) for classification errors.

Undertriage rate *α*	0	0.1	0.2	0.3	0.4

PLI	0.50	0.72	0.80	0.83	0.84

Overtriage rate *β*	0	0.1	0.2	0.3	0.4

PLI	0.74	0.74	0.74	0.74	0.74

**Table 7:** Inefficiency of the ALS index (IAI) for classification errors.

Undertriage rate *α*	0	0.1	0.2	0.3	0.4

IAI	0.40	0.70	0.82	0.86	0.88

Overtriage rate *β*	0	0.1	0.2	0.3	0.4

IAI	0.73	0.73	0.73	0.74	0.74

## Data Availability

The data used to support the findings of this study are available from the corresponding author upon request.
